# Comparison of a xeno-free and serum-free culture system for human embryonic stem cells with conventional culture systems

**DOI:** 10.1186/s13287-016-0347-7

**Published:** 2016-07-30

**Authors:** Dan Zhang, Qingyun Mai, Tao Li, Jia Huang, Chenhui Ding, Mengxi Jia, Canquan Zhou, Yanwen Xu

**Affiliations:** Reproductive Medicine Center, the First Affiliated Hospital of Sun Yat-sen University, 58 Zhongshan Road II, Guangzhou, Guangdong 510080 People’s Republic of China

**Keywords:** Human embryonic stem cell, Xeno-free and serum-free culture system, Chemically defined medium, Human foreskin fibroblast cells

## Abstract

**Background:**

Elimination of all animal components during derivation and long-term culture of human embryonic stem cells (hESCs) is necessary for future applications of hESCs in clinical cell therapy.

**Methods:**

In this study, we established the culture system of xeno-free human foreskin fibroblast feeders (XF-HFF) in combination with chemically defined medium (CDM). XF-HFF/CDM was compared with several conventional culture systems. The hESCs cultured in different media were further characterized through karyotype analysis, pluripotency gene expression, and cell differentiation ability.

**Results:**

The hESCs in the XF-HFF/CDM maintained their characteristics including typical morphology and stable karyotype. In addition, hESCs were characterized by fluorescent immunostaining of pluripotent markers and teratoma formation in vivo. RT-PCR analysis shown that the stem cell markers OCT3/4, hTERT, SOX2, and Nanog were present in the cell line hESC-1 grown on XF-HFF/CDM. Furthermore, the results of cell growth and expression of bFGF, Oct-4, and hTERT indicated that XF-HFF/CDM had better performance than human serum-matrix/CDM and XF-HFF/human serum.

**Conclusion:**

The comparison of different xeno-free culture conditions will facilitate clarifying the key features of self-renewal, pluripotency, and derivation and will shed light on clinic applications of hESCs.

## Background

Human embryonic stem cells (hESCs) can differentiate into various cell types and possess great potential for cell-replacement therapy including treatment for diabetes, cardiac infarction, and neurodegenerative diseases [1, 2]. The major challenge for the clinical application of hESCs are animal-derived products and undefined factors during in-vitro establishment and expansion of the cells. Nevertheless, hESCs are conventionally cultured on feeder cell layers, of which mouse embryonic fibroblasts (MEFs) are the most common and present several unknown animal-derived products [3]. The risk of graft rejection and immunoreactions will be increased greatly, demonstrating that a xeno-support system sets a limitation for the therapeutic potential of hESCs to a great extent [4–6]. Optimization and standardization of a fully defined xeno-free culture for hESCs is therefore urgent.

Xeno-free culture conditions continue to evolve to avoid exposure to animal-derived materials [7–9]. However, some issues including chromosomal abnormalities in hESCs arise due to more demanding growth conditions [10]. On the contrary, feeder-free cultures generally present a higher degree of spontaneous differentiation in comparison with conventional cultures, signifying that higher concentration of exogenous basic fibroblast growth factor (bFGF) is required and feeder-free derivation is not optimal for developing transplantable hESC derivatives [11].

Considerable progress has been made towards maintaining hESC cultures since human feeder cells were proposed [12]. Several researchers have proved the ability to culture hESCs on human feeder cells which were isolated from human tissues, including adult marrow tissues [13], fetal muscle, fetal skin, human placental fibroblasts [14], and human endometrial cells [15]. Some contributions have been made to derive and culture new human foreskin feeders (hFFs) [16–18].

Other groups have attempted to culture hESCs in chemically defined as well as xeno-free conditions. Some studies have claimed the defined media can retain hESCs successfully in their pluripotent state [19, 20], while other studies have contradicted this [21]. Chin et al. [22] described the defined StemPro and mTeSR1 media without feeder and the xeno-free HEScGRO medium with hFFs for hESC growth. Further efforts should be made to culture hESCs in anticipation of future applications.

In this study, we evaluated whether xeno-free human foreskin fibroblast feeders (XF-HFF) combined with chemically defined medium (CDM) could be successfully used in long-term maintenance of undifferentiated hESCs. At the same time, the characteristics of hESCs grown under these conditions were assessed after prolonged culture, including their unlimited and undifferentiated proliferative ability, maintenance of normal karyotype in long-term culture, and their developmental potential to differentiate into representative tissues of all three embryonic germ layers. In addition, the efficacy of this culture system was compared with other culture conditions with respect to cell growth and undifferentiated states.

## Methods

### Human material and ethics statement

The design and performance of this study conformed to the ethical standards of the Helsinki Declaration and our national legislation. This study was approved by the Medical Ethical Committee of The First Affiliated Hospital, Sun Yat-sen University. An informed consent form was signed by the participant or the legal guardian for children after receiving a written description of the study.

Human blood was obtained from healthy donors, infant foreskin from circumcised infants, and discarded human embryos from donors undergoing clinical in-vitro fertilization treatment. Patients who donated blood and foreskins were negative for HIV, hepatitis B and C, and syphilis.

### Preparation of xeno-free human foreskin fibroblast cells (XF-HFF)

Human serum (HS) and XF-HFF were prepared as described previously [1]. XF-HFF were cultured in 90 % Dulbecco’s modified Eagle’s medium (DMEM; Invitrogen, Carlsbad, CA, USA) supplied with 10 % HS, 1 % penicillin–streptomycin (PS; HyClone Laboratories, Inc., Logan, UT, USA). The medium was changed daily. XF-HFF were normally passaged by TrypLE Select (Invitrogen) at a 1:8–1:10 split ratio every 3–5 days.

For preparation of feeder layers, XF-HFF were treated with mitomycin C (Sigma, St. Louis, MO, USA) for 2.5 h, and the treated cells were reseeded at 75,000 cells/cm^2^.

### Culture media

Four culture systems were described in this study as follows: XF-HFF combined with CDM (HEScGRO; Chemicon, Billerica, MA, USA) (XF-HFF/CDM group), XF-HFF combined with 20 % HS medium (XF-HFF/HS group), HS-matrix coated on plates without feeder layers combined with CDM (HS-matrix/CDM group), and xeno-contained human foreskin fibroblast feeders (XC-HFF) combined with 20 % KnockOut Serum Replacement (KSR medium) (XC-HFF/KSR group). Two hESC lines (hESC-1, hESC-2) derived in our laboratory were propagated for 25 passages in the XC-HFF/KSR group, and were then transferred to the other three hESC culture systems.

### RT-PCR

Total RNA was extracted using the RNeasy kit (Qiagen, Valencia, CA, USA). Approximately 500 ng of RNA template was reverse-transcribed into cDNA using the SuperScript III First-Strand Synthesis System for RT-PCR (Invitrogen). The cDNA was amplified by PCR using the Accuprime Taq DNA polymerase system (Invitrogen). The housekeeping gene glyceraldehyde-3-phosphate dehydrogenase (GAPDH) was used as an internal positive control. PCR products were run on a 2 % agarose gel, stained with ethidium bromide and visualized by UV illumination.

The primers sequences were: octamer-binding transcription factor Oct-4, 5′-GAAGGTATTCAGCCAAAC-3′ and 5′-CTTAATCCAAAAACCCTGG-3′; Nanog, 5′-GATCGGGCCCGCCACCATGAGTGTGGATCCAGCTTG-3′ and 5′-GATCGAGCTCCATCTTCACACGTCTTCAGGTTG-3′; human telomerase reverse transcriptase (hTERT), 5′-CGGAAGAGTGTCTGGAGCAAGT-3′ and 5′-GAACAGTGCCTTCACCCTCGA-3′; SOX2, 5′-CGGAAAACCAAGACGCTCA-3′ and 5′-GCCGTTCATGTAGGTCTGCG-3′; GAPDH, 5′-GTCAGTGGTG GACCTGACCT-3′ and 5′-CACCACCCTGTTGCTGTAGC-3′.

### Karyotype analysis of hESCs

The genetic stability of hESCs grown in the xeno-free culture system was determined by examining the karyotype using a standard G-banding procedure. Briefly, cells were incubated with colcemid, trypsinized, and then resuspended. The samples were then fixed onto the prepared slides to make the chromosome spreads. The dried slides were baked for 90 min at 80 °C, treated with 0.05 % trypsin for 30 sec to 2 min, and then stained with Giemsa and Leishman’s solution.

### Teratoma formation

Teratomas were generated by intramuscular injection of undifferentiated hESCs (6 × 10^6^ cells) into the hind limb of severe combined immunodeficient (SCID) mice. The injected mice were sacrificed at 8 weeks post injection, and the resulting teratomas were fixed with 4 % paraformaldehyde and embedded with paraffin. The samples were then stained with hematoxylin and eosin, and observed under a light microscope.

### Growth curve analysis

hESCs were plated at a density of 40,000 cells per well and cultured in different medium. The number of cells was counted using a hemacytometer at days 3, 5, and 7 to obtain a growth curve.

### Flow cytometry

hESC cultures were rinsed with PBS and then incubated with 0.5 mM EDTA (Sigma) at 37 °C for 5–8 min. The dissociated cells were rinsed twice in PBS for further analysis. Single cells were then blocked with 10 % goat serum (HyClone) for 15 min. All staining procedures were performed using buffer containing 2 % goat serum, 0.1 % sodium azide (Sigma), and 2 mM EDTA in PBS. The blocked cells were incubated with primary antibodies (stage-specific embryonic antigen SSEA-4 or tumor rejection antigen TRA-1-60, 1:20; Chemicon, Temecula, CA) for 30 min at 4 °C followed by three washes in PBS. The cells were then probed with a 1:100 dilution of Alexa-488-conjugated goat F(ab′)_2_ anti-mouse immunoglobulin (Ig)G_3_ or phycoerythrin-conjugated goat F(ab′)_2_ anti-mouse IgM (both from Invitrogen) for 30 min in the dark at 4 °C. The cells were washed once and resuspended for analysis in staining buffer containing 1 μg/ml of propidium iodide (Sigma) to identify nonviable cells. Flow cytometry analysis was performed using a FACS Calibur flow cytometer (BD Bioscience, San Jose, CA, USA).

### Quantification of Oct-4 and hTERT mRNA

RNA was isolated from hESC populations using an RNeasy kit (Qiagen), according to the manufacturer’s protocol. Extracted RNA was subsequently treated with DNA-free DNase I (Ambion, Austin, TX, USA). Real-time PCR amplification was performed on an Applied Biosystems International 7500 Sequence Detection System (Applied Biosystems, Foster City, CA, USA). The TaqMan one-step RT-PCR master mix (Applied Biosystems) was applied with the following reaction conditions: RT at 48 °C for 30 min; denaturation and AmpliTaq gold activation at 95 °C for 10 min; and amplification for 40 cycles at 95 °C for 15 sec and at 60 °C for 1 min. All reactions were performed in triplicate. The Oct-4 sequences were designed by Applied Biosystems Primer Express software: forward primer, 5′-GCA ACC TGG AGA ATT TGT TCC T-3′; reverse primer, 5′-CCA CAC TCG GAC CAC ATC CT-3′; and probe, FAM-5′-CAG TGC CCG AAA CCC ACA CTG C-3′-TAMRA. The 18 s and hTERT probe and primers were purchased from Applied Biosystems. Relative quantification of gene expression between multiple samples was achieved by normalization against endogenous18S ribosomal RNA using the △△CT method of quantification (Applied Biosystems). Relative fold difference in gene expression was calculated as 2^–(ΔΔCT)^.

### Immunofluorescence

The characterization of hESCs grown in animal-free conditions was carried out by measuring the expression of specific pluripotency markers including: SSEA-3, SSEA-4, SSEA-1, TRA-1-60, and TRA-1-81. hESCs were washed once in PBS and fixed in 4 % paraformaldehyde (DGCS biotech) at room temperature for 15 min. The cells were then blocked in PBST (PBS containing 0.1 % Triton 100 (DGCS) and 0.25 % normal donkey serum (Jackson Immuno Research Laboratories, West Baltimore, USA)) at room temperature for 1 h. Primary and secondary antibodies were diluted in PBST. Cells were incubated with primary antibodies at 4 °C for 2 h, followed by three rinses and then incubation with secondary antibodies at room temperature for 1 h. Cells were then washed three times in PBS prior to imaging. Stained preparations were examined under a Nikon Eclipse TE-2000 U fluorescence microscope (Nikon, Tokyo, Japan).

### Statistical analysis

All data were obtained from three replicate experiments. Values from all experiments were expressed as the mean ± standard deviation (SD). Student’s *t* test and a chi-square test were employed to analyze the statistical differences between culture groups. The analyses were performed using SPSS software version 10 (SPSS Inc., Chicago, IL, USA). *p* < 0.05 was considered statistically significant.

## Results

### Characterizations of hESCs in the XF-HFF/CDM culture system

#### hESCs cultured in XF-HFF/CDM exhibit normal cell morphologies

**Fig. 1:**
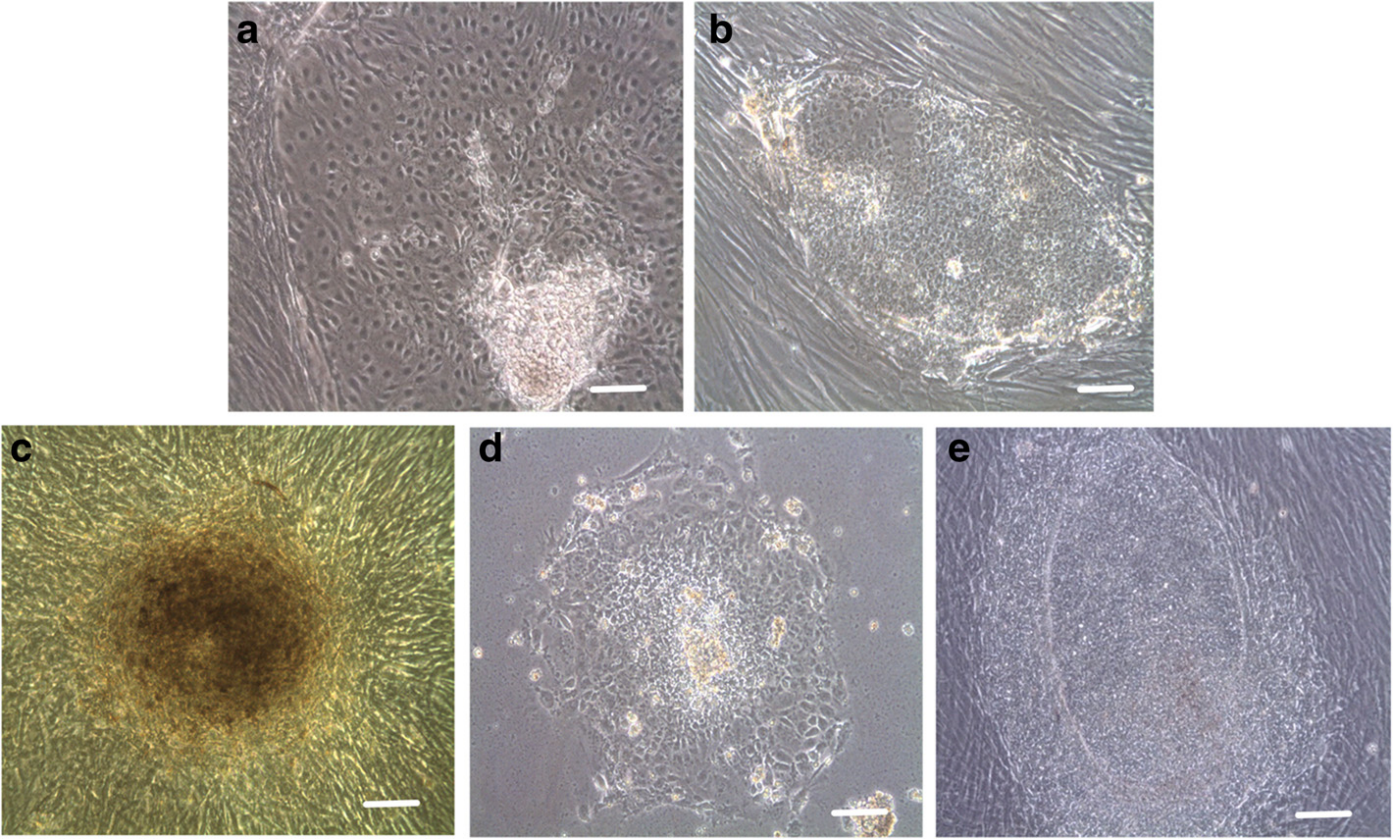
Morphology of hESC colonies cultured in different conditions. **a** hESC-1 showed a differentiated state after five passages cultured in XF-HFF/CDM conditions. **b** hESC-1 maintained an undifferentiated state cultured in XF-HFF/CDM conditions after 40 passages. **c** Morphology of hESC-1 cultured in XF-HFF/HS medium after five passages is elongated with a high nucleus/cytoplasm ratio, but a low propagation rate. **d** Morphology of hESC-1 cultured on HS-matrix in CDM medium after five passages is elongated with a low nucleus/cytoplasm ratio and propagation rate. **e** Morphology of hESC-1 cells on XC-HFF in KSR medium after 45 passages was still undifferentiated with a high nucleus/cytoplasm ratio and high propagation rate. Scale bar represents 50 μm

To determine whether hESCs grown in XF-HFF/CDM were maintained in an undifferentiated state, the morphology of hESC-1 cultured in XF-HFF/CDM was examined. As shown in Fig. 1a,b, the hESCs were found to suffer a series of morphological changes after transferring to the new environment. When the undifferentiated colonies were selected and passaged further, the clones gradually adapted to the culture system and regained undifferentiated morphology, tightly packed with sharp edges. The XF-HFF/CDM culture system was able to support maintenance of undifferentiated hESCs for more than 40 passages. The XF-HFF/CDM culture system can thus maintain hESCs in an undifferentiated state in the long term without losing their morphological characteristics.

#### hESCs cultured in XF-HFF/CDM express stem cell markers

**Fig. 2:**
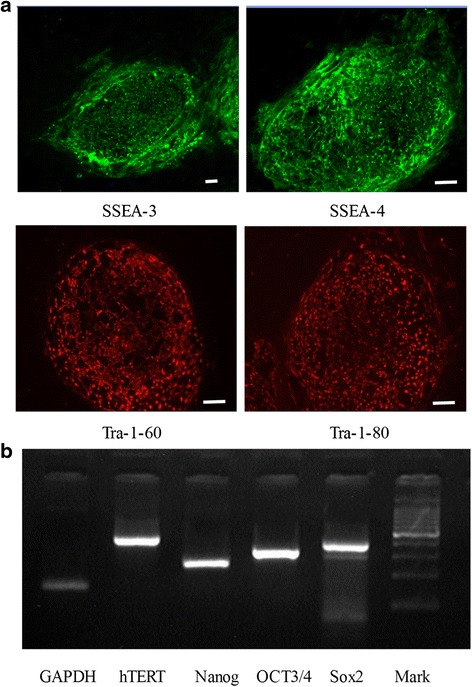
Expression of stem cell markers in hESC-1 grown on XF-HFF in CDM conditions after >30 passages. **a** Immunofluorescence staining with antibodies of stem cell markers: SSEA3 (*upper left*), SSEA4 (*upper left*), TRA-1-60 (*lower left*), and TRA-1-81 (*lower right*). **b** RT-PCR analysis of the expression of stem cell markers OCT3/4, hTERT, SOX2, and Nanog, with GAPDH as positive control. Scale bar represents 50 μm. *GAPDH* glyceraldehyde-3-phosphate dehydrogenase, *hTERT* human telomerase reverse transcriptase, *OCT* octamer-binding transcription factor, *SSEA* stage-specific embryonic antigen, *Tra* tumor rejection antigen

hESCs express stem cell markers that distinguish them from differentiated cells. To confirm that hESCs grown in the XF-HFF/CDM are undifferentiated, this was measured through indirect immunofluorescence staining. The results showed that hESC-1 grown in XF-HFF/CDM expressed the expected stem cell markers SSEA-3, SSEA-4, TRA-1-60, and TRA-1-81 (Fig. 2a). Expression of OCT3/4, hTERT, SOX2, and Nanog was further verified by RT-PCR analysis (Fig. 2b). These results indicated that the exogenous factors in XF-HFF/CDM were sufficient for hESC growth in an undifferentiated state.

#### Karyotype of hESCs cultured in XF-HFF/CDM

**Fig. 3:**
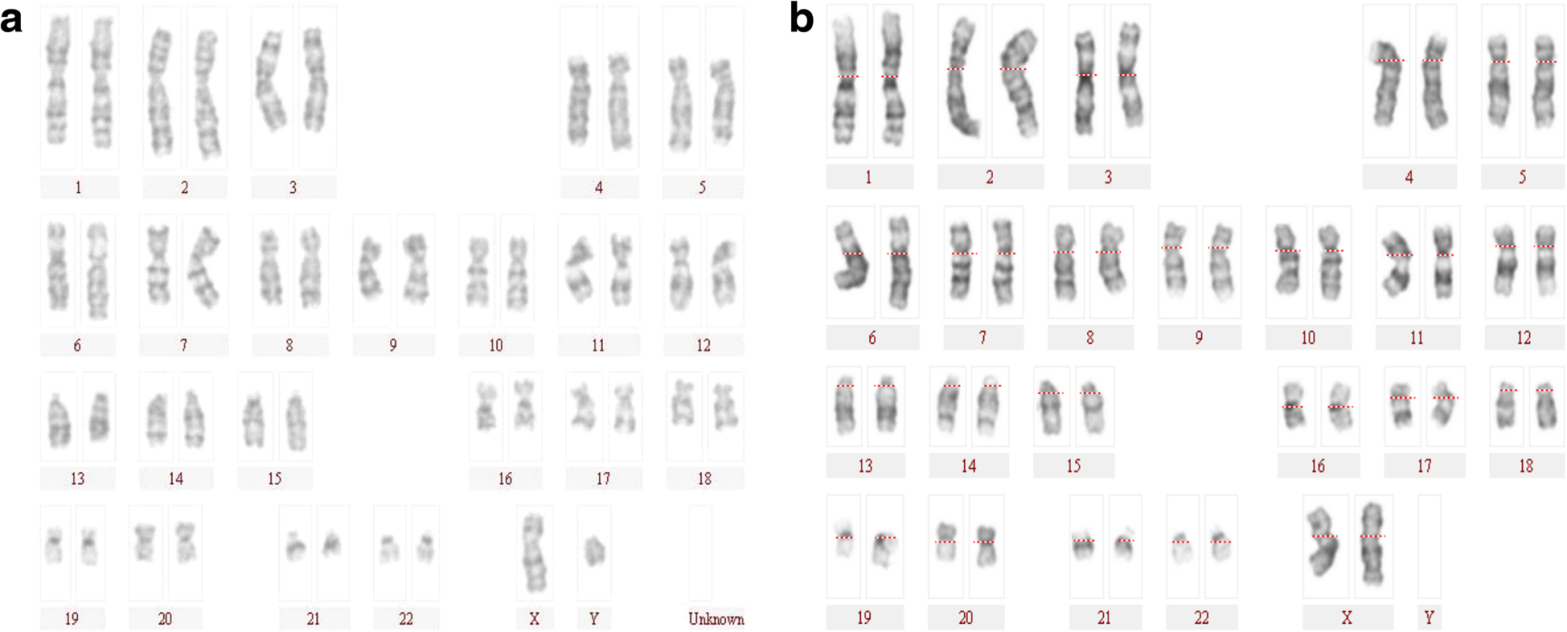
Genetic stability of hESCs cultured in XF-HFF/CDM. Karyotypes of **a** hESC-1 cells and **b** hESC-2 cells cultured in XF-HFF/CDM after >40 passages, respectively

Because we demonstrated that hESCs cultured in animal-free XF-HFF/CDM had normal morphological characteristics and expressed stem cell markers, we next determined whether these hESCs retained a normal chromosome complement because hESCs cultured in vitro may lose their genetic integrity after several passages. To examine the genetic stability of hESCs in XF-HFF/CDM, hESC-1 and hESC-2 cells were karyotyped after >40 passages. The results showed that the hESCs maintained their normal diploid karyotypes (46 XY and 46 XX, respectively) stably after more than 40 consecutive passages (Fig. 3a,b). No major translocations or other chromosomal changes were observed during this period.

#### hESCs cultured in XF-HFF/CDM are pluripotent

**Fig. 4:**
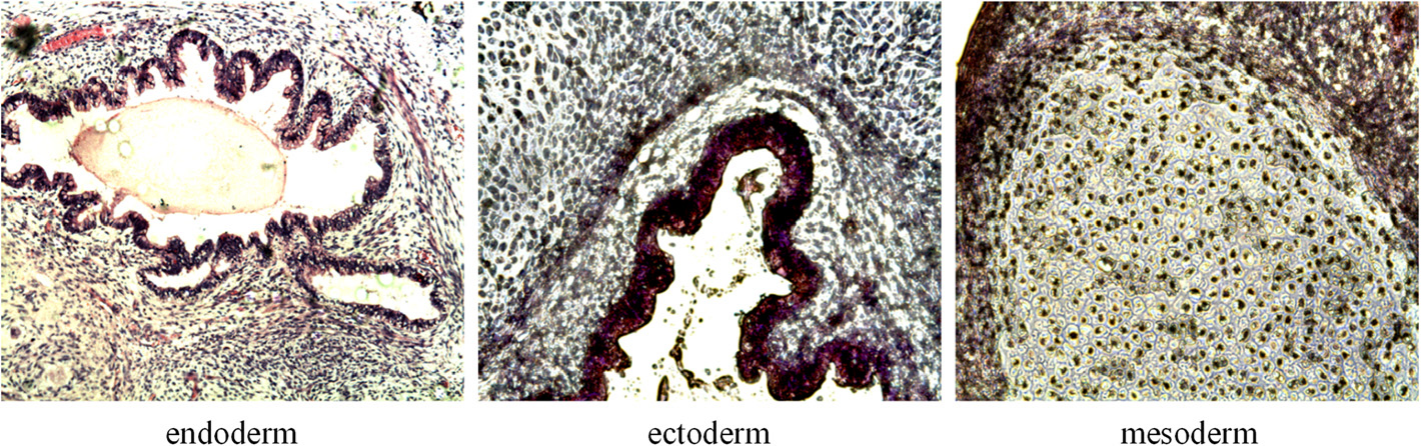
In-vivo analysis of the pluripotency of hESCs cultured in XF-HFF/CDM. hESC-1 cells were s.c. injected into the SCID mice. Sections of the resulting teratomas were stained with hematoxylin and eosin. Histological analysis of teratomas derived from hESCs cultured in xeno-free conditions after 35 passages. All three germ-layer-derived tissues were observed, including secretory epithelium (endoderm) (*left*, 10× magnification), skin epithelium (ectoderm) (*middle*, 20× magnification), and cartilage (mesoderm) (*right*, 20× magnification)

hESCs are pluripotent cells that can differentiate into the three major cell lineages: endodermal, ectodermal, and mesodermal. To further describe the differentiation potential of these hESCs grown in XF-HFF/CDM, we used teratoma formation to observe which tissues would develop from these cultures in vivo. Using histochemical analysis of teratomas after culturing for over 35 passages in the XF-HFF/CDM conditions, we observed the appearance of tissues representing all three germ layers – endoderm (secretory epithelium), ectoderm (skin epithelium), and mesoderm (cartilage) (Fig. 4). These tissues contained multiple cell types from each of the major cell lineages. Hence, the cells cultured in XF-HFF/CDM maintained their pluripotency in vivo.

### Comparison of different hESC culture systems

Recently, a xeno-free and feeder-free culture system based on HS-matrix and CDM or a cofeeder culture system based on xeno-free feeder cells and HS medium has been developed. To further access their culture efficacy, the characteristics of hESCs grown in XF-HFF/CDM were compared with XF-HFF/HS and HS-matrix/CDM. Two hESC lines were transferred from the original XC-HFF/KSR culture conditions to other three culture systems. As shown in Table 1, after transferring four passages the hESCs in XF-HFF/CDM adapted to the new environment quickly, and their survival rate was similar to that in XF-HFF/KSR. Furthermore, the differentiation rate was far lower than that of HS-matrix/CDM and XF-HFF/HS. These results demonstrate that it is easier to meet the requirements for hESC growth in the XF-HFF/CDM system.

**Table 1:** Culture efficacy of XF-HFF/CDM conditions and other culture conditions on the survival and differentiation rates of hESCs after transferring four passages

Group	Colony formation rate (%)^a^	Differentiation rate (%)^b^
XF-HFF/CDM(P_25+40_)	91.25 ± 2.22	6.00 ± 1.83
HS-matrix/CDM(P_25+40_)	26.5 ± 2.08*	85.00 ± 3.60*
XF-HFF/HS(P_25+40_)	77.75 ± 2.21*	86.00 ± 3.37*
XF-HFF/KSR(P_25+40_)	93.75 ± 2.22	5.50 ± 2.02

^a^Number of hESC colonies/total number of hESC colonies

^b^Number of hESC colonies with partial or whole differentiation/total number of hESC colonies

*p < 0.01, control groups versus new culture condition group

*CDM* chemically-defined medium, *hESC* human embryonic stem cell, *HS* human serum, *KSR* KnockOut Serum Replacement, *XF-HFF* xeno-free human foreskin fibroblast feeders

P25+40, defined as the embryonic stem cells which were established and cultured in XC-HFF/KSR culture system for 25 passages, then were shifted to the culture systems of experimental group and control group for 40 passages

#### Morphology of hESCs cultured with the other three culture media

Based on morphology, the colonies in XF-HFF/HS and HS-matrix/CDM became thinner and some lost their regular shapes and defined borders compared with that seen in XC-HFF/KSR by bright-field microscopy (Fig. 1c–e). In the XF-HFF/HS group and the HS-matrix/CDM group, hESCs lost their undifferentiated morphology and showed a high rate of differentiation and a low rate of colony formation (Fig. 1c,d). In addition, they were unable to maintain their undifferentiated states over six passages. The results indicated that XF-HFF/CDM had an advantage over XF-HFF/HS and HS-matrix/CDM in maintaining the pluripotency of hESCs.

#### Growth of hESCs cultured with the other three culture media

**Fig. 5:**
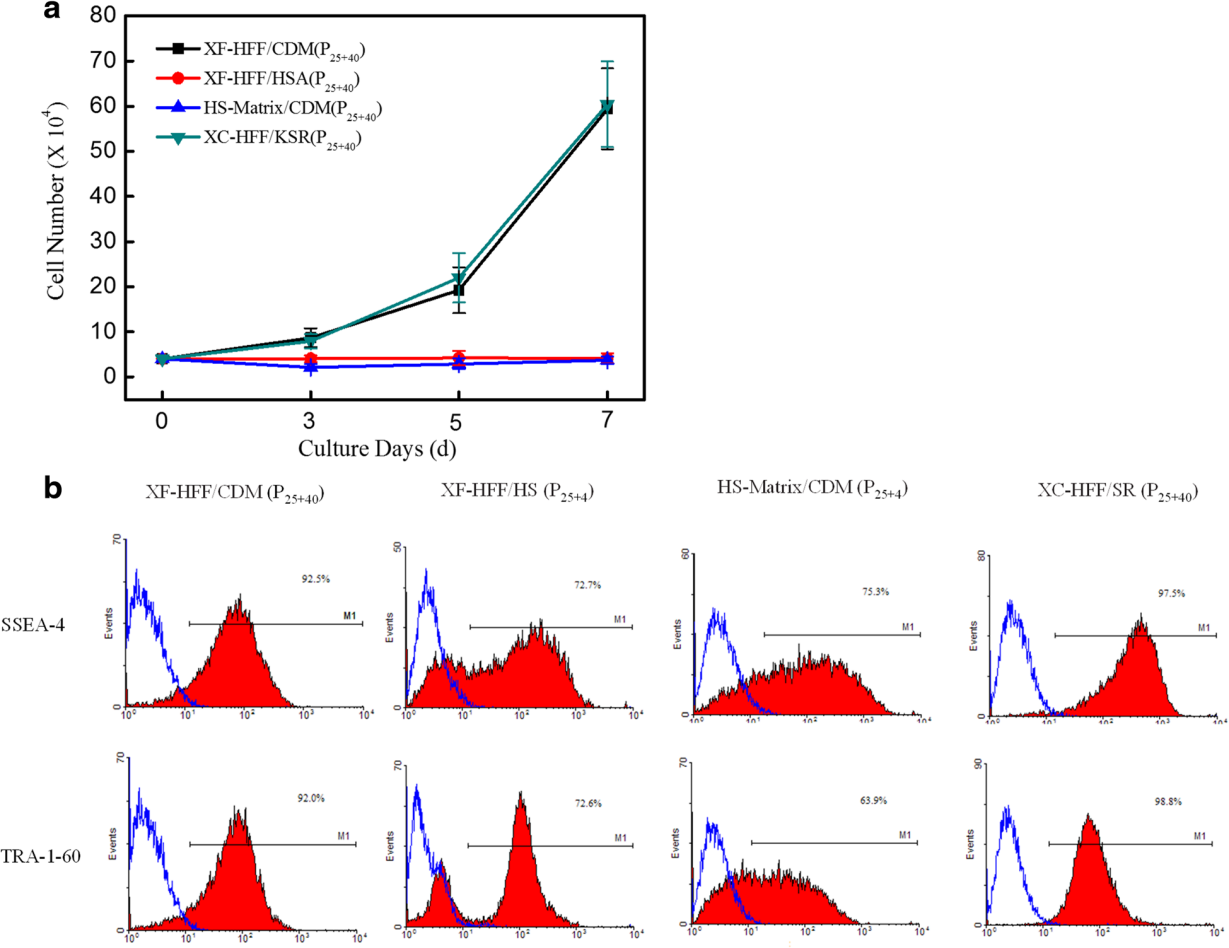
Comparison of different hESC culture systems. **a** Growth curve of hESCs cultured in four different conditions: 4 × 10^4^ cells from each culture condition were plated on day 0. Cell numbers were counted from triplicate wells at days 3, 5, and 7 after transferring from the initial XC-HFF/KSR condition. **b** Cells cultured in different conditions were analyzed on day 6 by fluorescence-activated cell sorting for SSEA-4 and TRA-1-60. *n* = 3. Error bars indicate SD. *CDM* chemically-defined medium, *HS* human serum, *KSR* KnockOut Serum Replacement, *SR* serum replacement, *SSEA* stage-specific embryonic antigen, *Tra* tumor rejection antigen, *XF-HFF* xeno-free human foreskin fibroblast feeders

To further understand the growth rate of hESCs, the cell number was calculated at days 3, 5, and 7 after transferring from the initial XC-HFF/KSR condition. As shown in Fig. 5a, the rate of cell proliferation in XF-HFF/CDM was obviously higher than that in XF-HFF/HS and HS-matrix/CDM, which had a similar trend to the growth curve in XC-HFF/KSR. The analysis verified that XF-HFF/CDM was superior to HS-matrix/CDM and XF-HFF/HS systems in supporting the growth of hESCs.

#### Undifferentiated states of hESC clones under different culture systems

The undifferentiated states of hESC clones under different culture systems were compared by analyzing the expression levels of SSEA-4 and TRA-1-60 by flow cytometry (Fig. 5b). Consistent with the morphologic observations, both CDM-cultured and KSR medium-cultured hESC clones on hFFs expressed high levels of undifferentiated surface marker expression. hESCs cultured in HS-matrix with CDM or HS medium on hFFs, however, downregulated these markers.

**Fig. 6:**
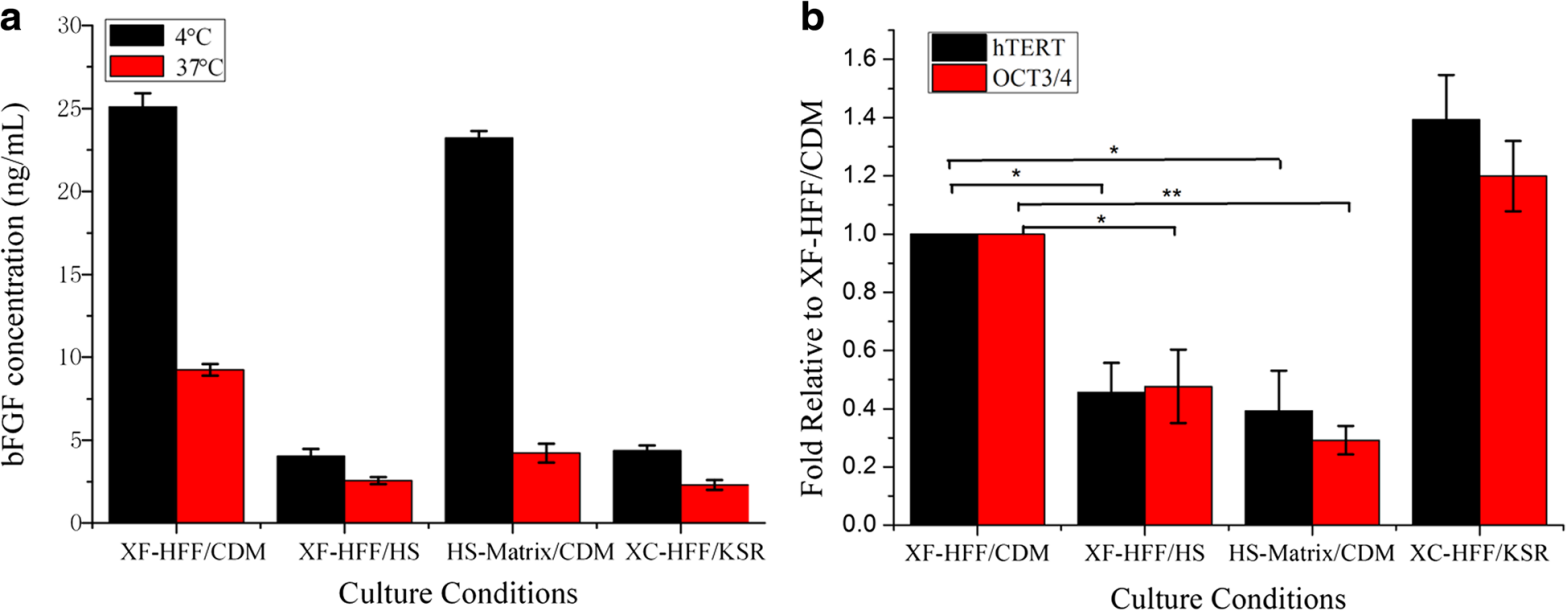
Expression levels of hESC markers under different culture systems. **a** Concentrations of bFGF in different hESC culture media were collected after overnight incubation at 4 or 37 °C. **b** Quantitative RT-PCR TaqMan analysis of Oct-4 and hTERT expression in hESCs maintained in various conditions. Samples were measured by ELISA for final FGF2 concentrations. Values with SDs are listed below bars in nanograms per milliliter. *n* = 3. *p < 0.05, **p < 0.01. Error bars indicate SD. *bFGF* basic fibroblast growth factor, *CDM* chemically-defined medium, *HS* human serum, *hTERT* human telomerase reverse transcriptase, *KSR* KnockOut Serum Replacement, *OCT* octamer-binding transcription factor, *XF-HFF* xeno-free human foreskin fibroblast feeders

We also examined the concentrations of bFGF in each hESC culture medium. After incubation at 37 °C for 24 h, we observed a significant reduction in bFGF levels in all cultures (*p* < 0.05), but the reduction was greater in medium from feeder-free conditions compared with that in medium from cultures with feeder cells (18.2 % vs 36.8–63.8 %) (Fig. 6a).

We performed quantitative RT (qRT)-PCR to confirm the expression levels of hESC markers. OCT-4 and hTERT were expressed in undifferentiated hESCs and downregulated upon differentiation. Figure 6b shows that hESCs in HS-matrix conditions or HS medium conditions expressed low levels of OCT-4 and hTERT. The expression level of the pluripotency marker OCT-4 under XF-HFF/CDM was twofold higher than that under HS-matrix conditions or HS medium conditions. Measurement of hTERT expression by RT-PCR in four culture systems showed that the changing trend was almost the same as that of OCT-4 (Fig. 6b). The hESCs maintained in the XF-HFF/CDM system therefore had relatively high levels of OCT-4 and hTERT, resembling undifferentiated hESCs maintained on XC-HFF/KSR.

## Discussion

As a renewable cell source for future regenerative medicine applications, the derivation, passaging, and culture of hESCs have attracted widespread attention [23]. To generate therapeutically safe and usable hESC derivatives for clinical cell treatment, all animal-derived materials which are a potential risk for infection transmitted by animal pathogens must be eliminated during the process [24].

The major sources of xeno-contamination are the presence of animal feeders and the use of either FBS or serum replacement (SR) in the culture medium [4]. Many groups have previously claimed successful culture of hESCs on hFFs and other feeders [7, 12, 14, 25–28]. Hovatta et al. [27] used commercially available human foreskin fibroblasts for hESC propagation. Crook et al. [29] proposed the proof of concept for the generation of six clinical-grade hESC lines using hFFs as feeder cells. hFFs isolated from infant foreskin have a distinct advantage over MEFs. In addition to the xenobiotic issue, hFFs can be cultured for up to 62 passages before senescence, while MFFs can only be propagated for five passages [7, 30]. Moreover, little ethical concern is considered over the acquirement of foreskin tissue for the generation of feeder cells.

Another obstacle to overcome was the presence of FBS or SR in the culture medium. Several studies described the use of HS as serum supplement to culture hESCs. However, it has been proved previously that commercial xeno-free SRs and human sera are inappropriate for long-term culture of hESCs [21]. Some attempts to use HS as a medium supplement failed due to spontaneous differentiation [12, 26, 31]. It is important to point out that HS may be involved in the interactions between secreted factors from the feeder cells. Therefore, it is highly desirable to employ defined components rather than complex mixtures [32–35]. The establishment of a chemically defined xeno-free and feeder-free culture system is advantageous for exact identification of factors secreted by feeder layers or signal pathways existing in them. Thus, xeno-free commercially available CDM was employed in the culture system.

Our data demonstrate that hESCs can be successfully derived and cultured long term in the XF-HFF/CDM system while maintaining their undifferentiated state. Furthermore, teratoma analysis demonstrated that hESCs maintain their pluripotency and differentiation potential, which were comparable with hESCs cultured in the conventional hESC culture medium. The use of CDM with hFF cells has been described previously [17]. On the basis of that work, we further presented the differences of culture systems for hESCs. We tested four culture systems and two hESC lines, and little variation was observed between hESCs cultured in CDM and KSR medium in maintaining undifferentiated states of hESCs. On the other hand, data obtained by flow cytometry and qRT-PCR demonstrated that feeder cells supported the growth of undifferentiated hESCs more efficiently than HS-matrix when cells were presented in CDM. The HS-matrix, which contains hyaluronic acid, fibronectin, and vitronectin, as well as other unknown human factors, supports the attachment and growth of undifferentiated cells [36]. Stojkovic et al. [37] suggested that HS used as a matrix maintained pluripotency and genomic stability of hESCs. Meng et al. [38] pointed that extracellular matrix isolated from foreskin fibroblasts has an advantage in the process of long-term xeno-free hESC culture. However, feeder-free cultures usually presented a higher degree of spontaneous differentiation than conventional culture along with a higher concentration of exogenous bFGF, which indicated that feeder-free derivation and culture were not suitable for developing transplantable hESC derivatives [11]. The results in this manuscript also proved that the HS-matrix/CDM system could not maintain the undifferentiated state and differentiated potential of hESCs. Although the molecular and developmental mechanisms controlling the pluripotency and differentiation of hESCs are largely unknown, feeder cells may provide a better, more stable environment, and thus they can secrete the unique proteins that participate in cell growth, extracellular matrix formation, and remodeling [1, 39].

The results demonstrated that CDM provided an effective replacement for HS medium, comparable with KSR medium, to support hESC self-renewal and pluripotency. This observation suggests that CDM and KSR medium culture conditions supply similar growth factors or signal pathways for hESC growth. CDM is more suitable than serum-containing medium for studies that aimed at identifying the exogenous signals required for undifferentiated hESC growth. XF-HFF/HS were unable to maintain undifferentiated of hESCs in long-term growth.

Exogenous bFGF has been identified as a key factor involved in self-renewal of hESCs [40–43], and some studies have indicated that FGF proteins are sensitive to thermal denaturation, while fibroblasts likely secrete either protease inhibitors or binding proteins that modulate bFGF stability [42, 44]. In our work, we found that bFGF was unstable in 37 °C culture conditions. Although the concentrations of bFGF declined in all culture conditions, they were higher in cofeeder conditions than in feeder-free conditions. The data indicated that fibroblast is likely to secrete some proteins which modulate the stability of bFGF [42], and also indicated that the stability of bFGF was not the only factor responsible for the successful culture of hESCs.

Lysophosphatidic acid (LPA) as the key lipid is a simple phospholipid mediator which plays an important role in the regulation of cell proliferation, migration, and survival of multiple cell types [45, 46]. Todorova et al. [47] proposed that LPA was involved in the expression of the Ca2^+^-dependent early response gene c-myc, which was a key gene implicated in ESC self-renewal and pluripotency. Therefore, LPA increased the proliferation rate of mESCs. Liu et al. demonstrated that LPA induced the expression of the erythroid biomarkers in cultured human hematopoietic stem cells (hHSCs) under plasma-free conditions. In addition, LPA was also reported to enhance osteogenic differentiation of human mesenchymal stem cells [48]. These results suggested that LPA may play a critical role in cell fate determination. Hence, we suspected that HFF-1 cells could secrete LPA to support the long-term culture.

## Conclusion

We have reported the development of a xeno-free and serum-free method for culturing hESCs. The hESCs in the established XF-HFF/CDM culture system maintained their properties, such as typical morphology, pluripotency, capacity to form three germ layers in vivo, and stable karyotype. The results show that the XF-HFF/CDM culture medium is applicable for further optimization of xeno-free establishment, culture, and differentiation of stem cells. Future studies should focus on the comparison of different xeno-free culture conditions and on validation of these conditions to demonstrate the key features of self-renewal, pluripotency, and derivation.

## Abbreviations

bFGF, basic fibroblast growth factor; CDM, chemically defined medium; DMEM, Dulbecco’s modified Eagle’s medium; ELISA, enzyme-linked immunosorbent-based assay; GAPDH, glyceraldehyde-3-phosphate dehydrogenase; hESC, human embryonic stem cell; hFF, human foreskin feeder; HS, human serum; hTERT, human telomerase reverse transcriptase; KSR, KnockOut Serum Replacement; LPA, lysophosphatidic acid; MEF, mouse embryonic fibroblast; OCT, octamer-binding transcription factor; qRT-PCR, quantitative RT-PCR; SCID, severe combined immunodeficient; SR, serum replacement; SSEA, stage-specific embryonic antigen; Tra, tumor rejection antigen; XC-HFF, xeno-contained human foreskin fibroblast feeders; XF-HFF, xeno-free human foreskin fibroblast feeders

## References

[1] Ellerström C, Strehl R, Moya K, Andersson K, Bergh C, Lundin K (2006). Derivation of a xeno‐free human embryonic stem cell line. Stem Cells..

[2] Yao S, Chen S, Clark J, Hao E, Beattie GM, Hayek A (2006). Long-term self-renewal and directed differentiation of human embryonic stem cells in chemically defined conditions. Proc Natl Acad Sci U S A..

[3] Choo AB, Padmanabhan J, Chin AC, Oh SK (2004). Expansion of pluripotent human embryonic stem cells on human feeders. Biotechnol Bioeng..

[4] Martin M, Muotri A, Gage F, Varki A (2005). Human embryonic stem cells express an immunogenic nonhuman sialic acid. Nat Med..

[5] Lu J, Hou R, Booth CJ, Yang SH, Snyder M (2006). Defined culture conditions of human embryonic stem cells. Proc Natl Acad Sci U S A..

[6] Skottman H, Hovatta O (2006). Culture conditions for human embryonic stem cells. Reproduction..

[7] Amit M, Shariki C, Margulets V, Itskovitz-Eldor J (2004). Feeder layer- and serum-free culture of human embryonic stem cells. Biol Reprod..

[8] Meng G, Liu S, Rancourt DE (2011). Synergistic effect of medium, matrix, and exogenous factors on the adhesion and growth of human pluripotent stem cells under defined, xeno-free conditions. Stem Cells Dev..

[9] Miwa H, Hashimoto Y, Tensho K, Wakitani S, Takagi M (2012). Xeno-free proliferation of human bone marrow mesenchymal stem cells. Cytotechnology..

[10] Draper JS, Smith K, Gokhale P, Moore HD, Maltby E, Johnson J (2004). Recurrent gain of chromosomes 17q and 12 in cultured human embryonic stem cells. Nat Biotechnol..

[11] Kim SJ, Cheon SH, Yoo SJ, Kwon J, Park JH, Kim CG (2005). Contribution of the PI3K/Akt/PKB signal pathway to maintenance of self-renewal in human embryonic stem cells. FEBS Lett..

[12] Richards M, Fong CY, Chan WK, Wong PC, Bongso A (2002). Human feeders support prolonged undifferentiated growth of human inner cell masses and embryonic stem cells. Nat Biotechnol..

[13] Cheng L, Hammond H, Ye Z, Zhan X, Dravid G (2003). Human adult marrow cells support prolonged expansion of human embryonic stem cells in culture. Stem Cells..

[14] Genbacev O, Krtolica A, Zdravkovic T, Brunette E, Powell S, Nath A (2005). Serum-free derivation of human embryonic stem cell lines on human placental fibroblast feeders. Fertil Steril..

[15] Lee JB, Lee JE, Park JH, Kim SJ, Kim MK, Roh SI (2005). Establishment and maintenance of human embryonic stem cell lines on human feeder cells derived from uterine endometrium under serum-free condition. Biol Reprod..

[16] Fletcher JM, Ferrier PM, Gardner JO, Harkness L, Dhanjal S, Serhal P (2006). Variations in humanized and defined culture conditions supporting derivation of new human embryonic stem cell lines. Cloning Stem Cells..

[17] Meng G, Liu S, Krawetz R, Chan M, Chernos J, Rancourt DE (2008). A novel method for generating xeno-free human feeder cells for human embryonic stem cell culture. Stem Cells Dev..

[18] Stacey GN, Cobo F, Nieto A, Talavera P, Healy L, Concha A (2006). The development of “feeder” cells for the preparation of clinical grade hES cell lines: challenges and solutions. J Biotechnol..

[19] Liu Y, Song Z, Zhao Y, Qin H, Cai J, Zhang H (2006). A novel chemical-defined medium with bFGF and N2B27 supplements supports undifferentiated growth in human embryonic stem cells. Biochem Biophys Res Commun..

[20] Li Y, Powell S, Brunette E, Lebkowski J, Mandalam R (2005). Expansion of human embryonic stem cells in defined serumfree medium devoid of animal-derived products. Biotechnol Bioeng..

[21] Rajala K, Hakala H, Panula S, Aivio S, Pihlajamaki H, Suuronen R (2007). Testing of nine different xeno-free culture media for human embryonic stem cell cultures. Hum Reprod..

[22] Chin ACP, Padmanabhan J, Oh SKW, Choo ABH (2009). Defined and serum-free media support undifferentiated human embryonic stem cell growth. Stem Cells Dev..

[23] Hoffman LM, Carpenter MK (2005). Characterization and culture of human embryonic stem cells. Nature Biotechnol..

[24] Mallon BS, Park KY, Chen KG, Hamilton RS, McKay RD (2006). Toward xeno-free culture of human embryonic stem cells. Int J Biochem Cell Biol..

[25] Inzunza J, Gertow K, Strömberg MA, Matilainen E, Blennow E, Skottman H (2005). Derivation of human embryonic stem cell lines in serum replacement medium using postnatal human fibroblasts as feeder cells. Stem Cells..

[26] Richards M, Tan S, Fong CY, Biswas A, Chan WK, Bongso A (2003). Comparative evaluation of various human feeders for prolonged undifferentiated growth of human embryonic stem cells. Stem Cells..

[27] Hovatta O, Mikkola M, Gertow K, Strömberg AM, Inzunza J, Hreinsson J (2003). A culture system using human foreskin fibroblasts as feeder cells allows production of human embryonic stem cells. Hum Reprod..

[28] Stojkovic P, Lako M, Stewart R, Przyborski S, Armstrong L, Evans J (2005). An autogeneic feeder cell system that efficiently supports growth of undifferentiated human embryonic stem cells. Stem Cells..

[29] Crook JM, Peura TT, Kravets L, Boesman AG, Buzzard JJ, Horne R (2007). The generation of six clinical-grade human embryonic stem cell lines. Cell Stem Cell..

[30] Meng GL, Zur Nieden NI, Liu SY, Cormier JT, Kallos MS, Rancourt DE (2008). Properties of murine embryonic stem cells maintained on human foreskin fibroblasts without LIF. Mol Reprod Dev..

[31] Koivisto H, Hyvärinen M, Strömberg AM, Inzunza J, Matilainen E, Mikkola M (2004). Cultures of human embryonic stem cells: serum replacement medium or serum-containing media and the effect of basic fibroblast growth factor. Reprod BioMed Online..

[32] Nakagawa M, Taniguchi Y, Senda S, Takizawa N, Ichisaka T, Asano K (2014). A novel efficient feeder-free culture system for the derivation of human induced pluripotent stem cells. Sci Rep-UK.

[33] Wang Q, Mou X, Cao H, Meng Q, Ma Y, Han P (2012). A novel xeno-free and feeder-cell-free system for human pluripotent stem cell culture. Protein Cell..

[34] Totonchi M, Taei A, Seifinejad A, Tabebordbar M, Rassouli H, Farrokhi A (2010). Feeder-and serum-free establishment and expansion of human induced pluripotent stem cells. Int J Dev Biol..

[35] Rajala K, Lindroos B, Hussein SM, Lappalainen RS, Mattila M, Inzunza J (2010). A defined and xeno-free culture method enabling the establishment of clinical-grade human embryonic, induced pluripotent and adipose stem cells. PloS ONE..

[36] Uhm JH, Dooley NP, Kyritsis AP, Rao JS, Gladson CL (1999). Vitronectin, a glioma-derived extracellular matrix protein, protects tumor cells from apoptotic death. Clin Cancer Res..

[37] Stojkovic P, Lako M, Przyborski S, Stewart R, Armstrong L, Evans J (2005). Human-serum matrix supports undifferentiated growth of human embryonic stem cells. Stem Cells..

[38] Meng G, Liu S, Li X, Krawetz R, Rancourt DE (2010). Extracellular matrix isolated from foreskin fibroblasts supports long-term xeno-free human embryonic stem cell culture. Stem Cells Dev..

[39] Prowse AB, McQuade LR, Bryant KJ, Van Dyk DD, Tuch BE, Gray PP (2005). A proteome analysis of conditioned media from human neonatal fibroblasts used in the maintenance of human embryonic stem cells. Proteomics..

[40] Vallier L, Alexander M, Pedersen RA (2005). Activin/Nodal and FGF pathways cooperate to maintain pluripotency of human embryonic stem cells. J Cell Sci..

[41] Xu RH, Peck RM, Li DS, Feng X, Ludwig T, Thomson JA (2005). Basic FGF and suppression of BMP signaling sustain undifferentiated proliferation of human ES cells. Nat Methods..

[42] Levenstein ME, Ludwig TE, Xu RH, Llanas RA, Van Den Heuvel‐Kramer K, Manning D (2006). Basic fibroblast growth factor support of human embryonic stem cell self-renewal. Stem Cells..

[43] Xu C, Rosler E, Jiang J, Lebkowski JS, Gold JD, O'Sullivan C (2005). Basic fibroblast growth factor supports undifferentiated human embryonic stem cell growth without conditioned medium. Stem Cells..

[44] Eiselleova L, Peterkova I, Neradil J, Slaninova I, Hampl A, Dvorak P (2008). Comparative study of mouse and human feeder cells for human embryonic stem cells. Int J Dev Biol..

[45] Chiang CL, Chen SSA, Lee SJ, Tsao KC, Chu PL (2011). Lysophosphatidic acid induces erythropoiesis through activating lysophosphatidic acid receptor 3. Stem Cells..

[46] Costa M, Sourris K, Lim SM, Qing CY, Hirst CE (2013). Derivation of endothelial cells from human embryonic stem cells in fully defined medium enables identification of lysophosphatidic acid and platelet activating factor as regulators of eNOS localization. Stem Cell Res..

[47] Todorova MG, FuentesE SB, Nadal A, Quesada I (2009). Lysophosphatidic acid induces Ca2+ mobilization and c-Myc expression in mouse embryonic stem cells via the phospholipase C pathway. Cell Signal..

[48] Liu YB, Kharode Y, Bodine PVN, Yaworsky PJ, Robinson JA (2010). LPA induces osteoblast differentiation through interplay of two receptors: LPA1 and LPA4. J Cell Biochem..

